# Training the Fetal Immune System Through Maternal Inflammation—A Layered Hygiene Hypothesis

**DOI:** 10.3389/fimmu.2020.00123

**Published:** 2020-02-11

**Authors:** April C. Apostol, Kirk D. C. Jensen, Anna E. Beaudin

**Affiliations:** Department of Molecular and Cell Biology, School of Natural Sciences, University of California, Merced, Merced, CA, United States

**Keywords:** hygiene hypothesis, hematopoietic stem cell, immunity, fetal-maternal, immune training

## Abstract

Over the last century, the alarming surge in allergy and autoimmune disease has led to the hypothesis that decreasing exposure to microbes, which has accompanied industrialization and modern life in the Western world, has fundamentally altered the immune response. In its current iteration, the “hygiene hypothesis” suggests that reduced microbial exposures during *early* life restricts the production and differentiation of immune cells suited for immune regulation. Although it is now well-appreciated that the increase in hypersensitivity disorders represents a “perfect storm” of many contributing factors, we argue here that two important considerations have rarely been explored. First, the window of microbial exposure that impacts immune development is not limited to early childhood, but likely extends into the womb. Second, restricted microbial interactions by an expectant mother will bias the fetal immune system toward hypersensitivity. Here, we extend this discussion to hypothesize that the cell types sensing microbial exposures include fetal hematopoietic stem cells, which drive long-lasting changes to immunity.

## Introduction

In this review, we will explore a body of work that demonstrates how maternal exposure to microbes during pregnancy has a significant impact on the development of the immune system in offspring. We will also review a growing body of literature that demonstrates how adult hematopoietic stem cells (HSCs) can sense diverse immune stimuli, thereby impacting the production, and sometimes function, of immune cell progeny. Furthermore, we will reconcile these two bodies of literature to suggest that maternal inflammation and infection are perceived by fetal HSCs to shape the immune system in the neonatal period and beyond. The consequences of these perturbations are underexplored but are likely to impact propensity for hypersensitivity disorders and resistance to certain infections as neonates. Thus, in addition to the passive transfer of maternally derived antibodies, the mother affords a separate mode of immune transfer, one that is driven by inflammation but acts upon a receptive HSC compartment during fetal development. The resultant training, or biased cell output, will have long ranging effects on neonatal and adult immune function. We argue that the hygiene hypothesis should encompass how microbial exposure during pregnancy fosters immune development through “training” immune output at the stem cell level.

## What is the Hygiene Hypothesis?

Thirty years have passed since the inception of the hygiene hypothesis, which attempted to reconcile an inverse correlation between birth order and incidence of allergic disease observed in British families (1). It was reasoned that in larger families with more children, communicable disease had a higher likelihood of being passed during early life. The immune education thereby afforded by enhanced microbial exposure in the youngest siblings was posited to be favorable for increased immune tolerance as compared to older siblings. Numerous similar observations have since been made for a variety of environmental conditions favorable to enhanced microbial exposure early in life, which include enrollment of newborns (6–11 months) in daycare centers (2) and growing up on farms (3). Conversely, the correlations between industrialization and conditions such as atopy (4) and type 1 diabetes (5) have been used to underscore the hygiene hypothesis. Industrialization in the latter half of the twentieth century in the United States accompanied major demographic changes which dramatically dropped infection intensities (6, 7). This shift included factors such as smaller household sizes (4.1 in 1930 to 2.5 in 2004 kids per family), fewer people living on farms (12.2% in 1950 to 2.6% in 1990 of the US population living on farms), increased household plumbing (50% in 1940 to 99% of houses with complete plumbing in 1990) (7), and prevalent antibiotic use and vaccination, all of which have worked to create a more “sterile” environment. According to the Centers for Disease Control, 1 in 10 kids in the United States will suffer from asthma and 1 in 4 suffer from some type of allergic disorder in Europe (8), leading some to label allergy as an epidemic (9). Even within countries from different continents, higher incidences of hypersensitivity disorders can be observed in urban compared to rural environments (10–12). Given the rapid increase in hypersensitivity disorders in the latter half the twentieth century, public commentary and interest in the connection between hygiene and hypersensitivity has not waned (13).

### Beyond Th1/Th2 Dichotomy

The primary focus of the hygiene hypothesis, from a mechanistic perspective, has been on immunological mechanisms that shift CD4 T helper (Th) cell differentiation profiles due to microbial exposures in the first years of life. The original view was that early infection skewed development of Th cell repertoire toward “proinflammatory” Th1 responses, which in general antagonize “allergy promoting” Th2 immunity and allergic disorders (14–16). The absence of early microbial exposure in overly hygienic environments would therefore bias the T cell repertoire toward Th2 responses, which are normally favored early in life (17–21). However, simple antagonism between Th1 and Th2 failed to explain why propensity for autoimmune diseases, induced by tissue-destructive Th1/Th17 pro-inflammatory immune responses and Th2-mediated atopic diseases, characterized by IgE production and mast cell degranulation to environmental antigens, were both increasing in western nations (6, 22). For example, house dust mite (HDM) allergen-specific IgE is observed at similar frequencies in populations from high and low-income countries, suggesting equal induction of antigen-specific Th2 responses in both locales. Yet, immediate hypersensitivity allergic skin reactions to HDM is several-fold greater in the westernized countries, suggesting immunological tolerance was readily achieved in low-income settings (23). This led to the hypothesis that repeated infections support a state of immune tolerance by inducing an “immune regulatory network” underpinned by the function of T regulatory cells and immuno-suppressive cytokines, like TGF-β and IL-10 (23). Additionally, hypersensitivity disorders are not simply driven by aberrant T helper cell responses, but also by multiple cells of the innate immune system including innate lymphoid cells (ILCs) (24, 25), tissue resident macrophages (26), and epithelial cells (27), all of which potentially serve as targets for immune modulation by microbial infection. Importantly, many of these cells have early developmental origins, and as we will explore later, are likely to be targeted by inflammatory cues during *in utero* development.

### Which Microbes or Stimuli Are Missing in a Hygienic Environment?

When considering what type of microbial interactions may be missing in hygienic environments of the west, both helminths and the microbiota have garnered the most attention. The inverse correlation between helminth infection rates and hypersensitivity disease intensity in tropical locals has long been suspected as a causal in nature (5, 28, 29). In mouse models, helminth infections or their products can suppress experimental autoimmune encephalomyelitis (EAE) induction (30, 31), collagen-induced arthritis (32), CD8 T cell immunity to viruses (33, 34), and allergy (35, 36). Among multiple strategies (37), helminths use excreted products (38) to down-modulate immune responses including specific induction of T regulatory cells through the TGF-β pathway (39), blocking TLR-induced DC maturation thereby favoring Th2 development (40–43), suppressing ILC2 activation by inhibiting epithelial release of IL-33 (44) and induction of alternative macrophages (45). In humans, profiling of children exposed to helminths revealed the strong presence of critical immunomodulatory cytokines, including IL-10 (46) and enhanced frequencies of regulatory T cells in the blood (47). Together, these data support a role of helminths as immune modulators (48).

Helminths are not the only microbial interaction capable of eliciting immune tolerance (49). Even in the west, where helminth infections are less frequent if not rare, Italian cadets seropositive for orally acquired pathogens such as *Toxoplasma gondii, Hepatitis A*, and *Helicobacter pylori*, but not for pathogens acquired by different routes, were much less likely to have atopic disease, especially when seropositive for at least two (50). Children on farms are exposed to a higher diversity of bacteria and fungi species, and these exposures correlated with lower atopic disease (51). In this setting, childhood exposure to gram negative endotoxin appears to be an important component of atopic disease protection (52). Certainly, exceptions have been reported for the role of microbial diversity and endotoxin exposure on atopy (53) and in several cases, helminth infections can exacerbate hypersensitivity disease (54), illustrating that the hygiene hypothesis cannot serve as a generic explanation for all inflammatory diseases with complex etiologies. However, as noted in both farm environments and mouse models, the microbial environment experienced early in life is an important factor driving multiple immune disease outcomes.

In addition to pathogens, it is now appreciated that microbial composition of the neonatal gut is an important variable impacting relative risk for atopic disease (7), prompting several revisions to the hygiene hypothesis best stated as a “microbial diversity hypothesis” (53). The microbiome of the mother has a direct impact on the infant microbiome, and maternal-derived sources include exposure to the vaginal canal (55) and breast milk (56). Cesarean births, formula feeding, and disruption of the infant microbiota by antibiotic use by both mother (57) and child can contribute to aberrant microbial colonization of the infant gut and distinctly impact bacterial diversity (58). These disruptions to microbial seeding are correlated with increased susceptibility to obesity (57), asthma (55, 59), and atopic dermatitis among others (60). There is now evidence that early colonization of the airway microbiome can be modulated at birth (61), and this may also impact disease outcomes. For example, at birth, both term and preterm infants displayed a more diverse airway microbiome compared to older preterm infants with established bronchopulmonary dysplasia (62), while asymptomatic colonization with *Streptococcus* in the infant nasopharynx during the first year of life was found to correlate with increased asthma susceptibility (63). Finally, there has been considerable debate as to the existence of a placental microbiome and whether it could influence offspring immunity. Studies utilizing DNA sequencing (64) and culturing of placental tissues and amniotic fluid (65) indicate that infant gut colonization is initiated *in utero*. However, recent work has demonstrated that placental contamination through labor or even laboratory regents accounted for a majority of identified bacteria with the exception of group B Streptococcus (66). The diversity of the microbiome during early life no doubt shapes the trajectory of the immune system, but the cellular mechanisms and signals that influence early life immune training are not well-established.

## Hygiene Hypothesis Revisited—A Prenatal Window Intersected By Fetal Hematopoiesis

At the root of the hygiene hypothesis is the concept of a sensitive or “critical” period of development, during which the phenotype of the adult immune system can be shaped by extrinsic or intrinsic inputs. While the hygiene hypothesis has mostly considered early postnatal exposure, accumulating evidence suggests that this critical window extends prenatally. For example, exposure to farm animal shed *during pregnancy* is also a major factor in modifying immune function and reducing risk of allergic disease in offspring (3) and correlates with enhanced induction of cord blood T regulatory cells (67). When modeled in mice, in a maternal TLR-dependent manner, endotoxin exposure during pregnancy ameliorates allergic sensitivities in the progeny of exposed dams (68) and increases tracheal T-reg percentages (69). Conversely, anti-helminth therapy given during pregnancy correlates with increased allergic eczema in newborns, suggesting immune training afforded by the maternal environment impacts immunity to unrelated antigens (70). Similarly, maternal antibiotic exposure during early pregnancy is associated with an increased risk of allergic disease, although this association could also be explained by greater maternal susceptibility to infection (71). Collectively, these and similar observations have suggested a revision to the hygiene hypothesis, mainly that the critical window be extended into the womb (72). Below, we consider evidence to suggest that susceptibility to hypersensitivity and autoimmunity may be driven by fetal hematopoietic stem cells that sense maternal inflammatory cues, resulting in an altered immune trajectory.

### Prenatal Exposure to Infection Shapes Early Immunity

**Figure 1:**
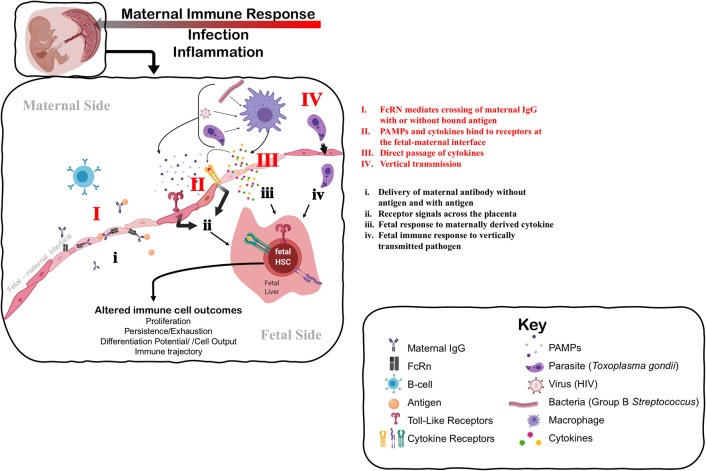
Understanding the mechanisms that impact fetal immune development in response to maternal immune perturbation. The maternal immune response to infection and inflammation is necessarily perceived by the fetus at the fetal-maternal interface. The fetus may perceive the maternal immune response through: I. Direct transfer and exposure to maternal antigen (i) via antibody-antigen complexes mediated by neonatal Fc receptor (FcRn); II. Receptors on the maternal side responding to PAMPs (Pathogen-Associated Molecular Patterns) produced by pathogens and maternal cytokines that signal to the fetal side (ii) through TLRs (Toll-Like Receptors) and specific cytokine receptors, respectively; III. Direct passage of cytokines across the fetal-maternal interface interacting directly with receptors on the fetal side that may evoke a different cellular response on the fetal side (iii); or IV. Vertical transmission of infection from mother to fetus, causing immune cells to directly perceive and respond to infection (iv). Fetal HSCs can respond to these signals of maternal infection and inflammation by direct changes to their function, including changes in cell proliferation or quiescence that alter the persistence of progenitors, and changes to differentiation potential and cellular output. Such changes at the HSC level can alter the trajectory of the immune system in a way that impacts immune homeostasis and function throughout the lifespan. Figure created using Biorender.com.

A growing body of evidence suggests that maternal exposure—both to non-infectious stimuli and infectious microbes—shapes the fetal and subsequent neonatal immune response (Figure 1). The most studied mode of influence of the maternal immune system on fetal and neonatal immunity is the transfer of maternally derived immunoglobulin (Ig) to the offspring, or passive immunity (Figure 1I). This transfer can occur both prenatally through the placenta, or postnatally in breastmilk, mediated by the neonatal Fc receptor, FcRN (73), and provides critical protection to the newborn. Importantly, transplacental transport of maternal IgG-antigen complexes by FcRn can also result in direct “priming” of antigen-specific immune responses in fetal cells (74–76) (Figure 1i). The FcRN mechanism may underscore antigen-specific responses to parasitic antigens by newborn lymphocytes in the context of maternal infection with schistosomiasis, placental malaria, Chagas' disease, and HIV (77). Importantly, fetal infection itself (Figure 1IV) is not a requirement for *in utero* priming of the fetal immune system (77). Indeed, multiple human studies and experimental systems have reported lymphocyte proliferation or cord blood IgM reactivity to vaccine antigens that are present at birth from vaccinated mothers (78). Maternal transfer of antigen can induce the presence of antigen-specific Tregs (79). However, whether these maternal-derived antigen specific fetal Tregs that are generated in the fetal thymus (nTregs) or periphery (pTregs) is unclear (80).

Beyond antigen-specific response driven by maternal-mediated antigen exposure, the maternal immune response *also evokes antigen-non-specific responses* in the developing fetal immune system (e.g., Figure 1ii,iii). For example, maternal vaccination during pregnancy is broadly associated with reduced mortality to unrelated pathogens in offspring (78), suggesting that generalized and protective neonatal immune responses can be elicited. Conversely, prenatal exposure to infection in the context of HIV, malaria, and a cross section of helminth infections correlates with increased susceptibility to diverse infections in neonates and poorer responses to vaccination postnatally (77). Whether the outcome of maternal exposure is protective or deleterious may depend on the nature of the maternal immune response, severity of disease, or mechanism of action. As an example, uninfected infants born to mothers with more advanced HIV disease experience a greater risk of perinatal morbidity and mortality (81). On the other hand, adverse pregnancy and infant outcomes associated with maternal infection can be attenuated if the maternal inflammatory response is experimentally controlled by administration of a microbial immunomodulatory agent (82). These data suggest that the degree of maternal inflammation can directly influence fetal outcomes.

Mounting evidence suggests that the fetal innate immune system can be “trained” during pregnancy (83, 84), by which maternal infection induces generalized and persistent changes to the function of the fetal innate immune system. Some of the best evidence for this comes from studies of infants born exposed to but uninfected with HIV [for review see (85)]. *In utero* exposure, but not vertical transmission with HIV, results in enhanced neonatal cytokine profiles of monocytes stimulated with various TLR agonists (86). Similarly, infants exposed prenatally to malaria demonstrated reduced basal levels of innate cytokines in cord blood, but higher responsiveness to stimulation with specific TLR agonists (87, 88). Human infants exposed to Hepatitis B Virus (HBV) *in utero* have higher levels of anti-viral cytokines in cord blood and exhibit evidence of greater activation and maturity of monocytes (89). Maternal vaccination during pregnancy can also heighten the innate immune response in offspring, as evidenced by an association between maternal *Bacille Calmette-Guérin* (BCG) scar size and infant pro-inflammatory cytokine production elicited by TLR stimulation (90). Training of the innate immune system in infants that occurs in the absence of vertical transmission underscores the ability of the fetal immune system to respond in an indirect manner to maternal infection or inflammation (Figure 1).

The neonatal adaptive immune response may also be intersected by a fetal trained innate immune system, and its response would depend on how and to what degree the developing innate immune system is evoked by maternal inflammation. For example, when mothers are infected with helminth pathogens during pregnancy, their newborns generally exhibit blunted Th1 responses to BCG vaccination (91, 92) and lower antibody titers following diphtheria toxin (DT) and Haemophilus influenzae (Hib) vaccination (93). In some cases, enhanced leukocyte production of the immunosuppressive cytokines, TGF-β and or IL-10, when stimulated with homologous vaccine antigen (i.e., “recalled”), correlates with poor vaccine responses due to antenatal helminth infections (91, 94). Additionally, HIV exposed but uninfected newborns have blunted humoral responses to measles (95), BCG (96), TT, and hepatitis B vaccination (97), and antenatal malaria also correlates with reduced neonatal vaccine responses to Hib and DT (93). In contrast, maternal infection with the kinetoplastid *Trypanosoma cruzi*, causes heightened neonatal adaptive immune response to BCG vaccination (98). Thus, antigen-non-specific changes to fetal immunity may additionally impact the adaptive immune response and trajectory toward immune homeostasis in newborns and possibly adults.

### Unknown Mechanisms of Fetal Immune Training by Maternal Inflammation

The mechanism underlying the response of the fetal immune system in the absence of overt fetal infection is unknown, and how indirect “training” of the fetal immune system by maternal infection or exposure occurs is unclear and understudied (Figure 1). One possible explanation for trained fetal immunity could be the direct passage of maternal cytokines or other inflammatory mediators into fetal circulation, which then stimulate the fetal immune system (Figure 1III). Determining whether maternal cytokines cross the placenta in humans during gestation is extremely challenging; *ex vivo* experiments with full-term human placenta suggests that transfer of cytokine across the placenta is limited at later developmental stages (99, 100). Nonetheless, evidence from rodent models suggests that some cytokines can cross the placenta earlier in gestation (101, 102), and subsequently modulate the neonatal response to infection (103). Dahlgren and colleagues demonstrated that transplacental passage of I^125^-labeled IL-6 was considerably higher at mid-gestation [embryonic day (E) 11–13] as compared to late gestation/near term (E17-19), suggesting that a less mature placenta may be more permeable to maternal cytokines (101). TLR ligands for specific pathogens were also recently shown to cross the mouse placenta at mid-gestation (E15) and directly impinge upon fetal cells; however, a direct effect on fetal immune cells was not described (104). Whether other TLR ligands can cross the placenta and directly elicit a fetal immune response has not been determined. In general, we know very little about how maternal cytokines or inflammatory mediators might induce the production or release of *different* cytokines on the fetal side (Figure 1ii,iii). Finally, vertically transmitted pathogens may directly stimulate fetal immune responses *in utero* (Figure 1IV,iv). Further investigation of the role of maternal cytokines and other inflammatory mediators in the direct induction of a fetal immune response, and the nature of that response, is warranted.

Another alternative explanation is that the fetus could respond indirectly to inflammation of or impingement on placental function caused by maternal infection (Figure 1II). Chorioamnionitis, an infection of the placenta typically caused by normally non-pathogenic microbes, drives systemic changes to the fetal immune system, including cytokine production and lymphocyte polarization (105). Importantly, fetal cytokine production has been observed in the absence of overt amniotic infection in a macaque model of Group B streptococcal-induced chorioamnionitis (106), suggesting that the fetus can respond directly to other signals outside of the fetal unit. Maternal viral infection of the placenta can also evoke fetal cytokine production in mice in the absence of fetal infection (107). Recent evidence from studies of cord blood in pre-term human infants suggests that inflammation at the maternal-fetal interface primes fetal lymphocytes to produce more inflammatory cytokines, including TNF-α and IFN-γ, in pre-term infants (108). Genetic dissection of the contribution of the fetal response to placental malaria recently revealed the requirement for fetal innate immune signaling in the control of placental malarial infection (109). Thus, the fetal immune system may respond to the consequences of maternal inflammation, as opposed to or in addition to a direct response to maternal inflammatory mediators.

Beyond inflammation and infection, growing evidence also suggests that the maternal microbiome can directly influence fetal immune development and function *in utero*. Although direct movement of maternal microbes to the placenta or fetus causes fetal demise, indirect exposure via microbial metabolites can influence fetal immune development. Limited gestational exposure to maternal *E. coli* colonization resulted in specific changes to fetal innate immune compartments, including gut Type III innate lymphoid cells (ILC3s) and mononuclear cells (110). Exposure may be dependent on maternal antibody-bound microbial molecules but could also be transmitted via direct exposure to microbial metabolites. For example, short chain fatty acids (SCFA), a microbial byproduct, can directly enter fetal circulation and influence fetal immune cell production, function, and ultimately offspring immunity (111). For example, SCFAs have been shown to influence susceptibility to allergic airway disease in adulthood by directly affecting adult hematopoiesis (112). Most recently, SCFA supplementation during pregnancy was also shown to rescue thymic and T-cell developmental defects in a mouse model of pre-eclampsia (113). In addition to the direct influence of the maternal microbiome on the infant microbiome, which ultimately influences neonatal immunity, direct or indirect exposure to metabolites *in utero* may also direct the prenatal immune response.

Activation of the fetal immune system in the context of maternal inflammation, infection, or exposure provides evidence that *in utero* exposure can directly evoke a fetal immune response (Figure 1). One outstanding question is whether or how a fetal immune response evoked during gestation translates into persistent changes in immune function into the neonatal period and beyond. Although fetal lymphocytes are generally considered to be long-lived immune cells, fetal and neonatal immune cells are eventually replaced by more mature immune cells over the course of postnatal development. In this case, how does *in utero* exposure imprint itself on the adult immune system? Below we consider a reframing of immune development to better understand how *in utero* exposure might have a long-term impact on immunity across the lifespan and particularly on vulnerability to hypersensitivity disorder.

### Fetal Immune Development Produces Tolerogenic Cells

Until recently, immune development has been perceived as relatively linear in nature: as the organism was exposed to and “experienced” pathogen, the immune system “matured” in tandem. Almost 30 years ago, however, Leonard Herzenberg described Ly5+ B1-B cells in adults that had distinct functions as compared to adult B cells and were produced specifically during fetal development (114). Since this initial discovery, increasingly sophisticated molecular and genetic approaches have led to an ever-growing list of specialized immune cells derived from fetal precursors. These include other subsets of innate-like lymphocytes—including γδ-T-cells (115–120), innate lymphoid cell subsets (ILCs) (121) and distinct T-cell subsets (122–124) as well as specific myeloid cells, such as tissue resident macrophages (125, 126) and mast cells (127). Importantly, many of these fetal-derived immune cells have been shown to persist across the lifespan of the animal, mostly independent of adult bone marrow (BM) hematopoiesis. Convincing evidence for a fetal origin of these specific immune cell compartments extend from (1) transplantation experiments revealing the enhanced or inclusive capability of fetal cells to reconstitute these compartments relative to adult BM cells (128–131), (2) parabiosis experiments revealing the minimal contribution of adult BM-derived hematopoiesis to these compartments under steady-state conditions (115, 129, 132, 133), or (3) fate-mapping or *in vivo* bar-coding experiments that have definitively shown the sustained contribution of fetal precursors to these populations in adulthood (115, 121, 125, 126, 131). The discovery of fetal-derived immune cells that persist and contribute to adult immunity with minimal contribution from adult BM hematopoiesis confirms that immune development is far from linear, and suggests that the phenotype of the adult immune system can be shaped from fetal development onwards.

A pivotal shift in immune function occurs at birth as the fetal immune system must switch from tolerogenic of the maternal environment to responsive to the external environment. This shift dictates the generation of immune cells with distinct functionality. In comparison to adult BM-derived immune cells, many fetal-derived immune cells recognize self- or commensal antigens, and function at the boundary of innate and adaptive immunity. They straddle a subtle functional balance as both mediators of tolerance and rapid responders to infection. For example, innate-like lymphocytes, including B1-B cells, γδ-T-cells, and innate lymphoid cells (ILCs), are rapid responders bearing either non-specific, germ-line encoded antigen receptors (B1-B cells, γδ-T-cells) or no antigen receptors (ILCs) that release natural antibody or cytokine in response to pathogen. Fetal-derived myeloid cells are mostly “tissue-resident,” and have unique functions in tissue homeostasis within their resident tissues. As these immune cells take up residency in their respective tissues across ontogeny, they both educate and are educated by the tissue microenvironment (134). For example, tissue-resident Kupffer cells in the liver function in iron recycling (135), and microglia are critical for synapse pruning during development (136). Thus, the establishment of these functionally distinct cell types from fetal precursors during development has critical implications not only for adult immune function, but also for normal tissue function and homeostasis across the lifespan.

Due to the specific functional attributes of fetal-derived immune cells in maintaining tolerance and tissue homeostasis, it is not entirely surprising that their dysregulation is implicated in disorders of tolerance, such as asthma and autoimmunity. For example, dysregulation of innate-like B cells and innate-like marginal zone B-cells has been observed in humans with, and mouse models of, autoimmune diabetes (137–139). B1-B cells have been specifically implicated as drivers of pathogenesis in autoimmune diabetes by producing IgG specific to self-DNA that promote inflammatory immune complexes in pancreatic islets (140). Similarly, activation of B1-B cells has been shown to promote pathogenesis in a variably penetrant mouse model of lupus (140–143). IL17-producing gamma-delta T-cells, including those of fetal origin, accumulate in a wide variety of autoimmune diseases including autoimmune encephalitis, psoriasis, and arthritis, where they are thought to enhance the adaptive response during autoimmunity (144–146). More recently identified innate lymphoid cell subsets have been similarly implicated in asthma and allergic diseases. Since the discovery of their importance in Type II immunity (147, 148), ILC2s have been shown to be crucial players in the development of allergic asthma (149, 150). Both ILC2s and ILC3s have been implicated in maintenance of gut homeostasis, and as cellular targets in inflammatory bowel disease (151, 152). Thus, disruption of fetal immune development may have distinct consequences for adult immunity by perturbing the establishment and function of immune cells that function at the boundary of tolerance and tissue homeostasis.

### Layered Immune Development Is Underscored by Transient Blood Progenitors

The generation of distinct immune cells during fetal development is driven from a series of discrete, transient hematopoietic progenitors that arise across multiple anatomical sites during ontogeny. The first wave of hematopoiesis occurs in the extraembryonic yolk sac, in so-called “blood islands” derived from endothelial cells that undergo endothelial to hematopoietic transition (EHT) to generate the first blood cells (153). These “primitive” blood cells consist primarily of large nucleated red blood cells, that meet that oxygenation needs of the early embryo, and primitive macrophages (154). Subsequently, more mature progenitors arise both in the yolk sac and in the developing aorta region, with increasingly diverse lineage potential (155). The first so-called “definitive” hematopoietic stem cells (HSCs), capable of replenishing the blood system after adoptive transfer into an irradiated adult recipient, arise in the developing aorta region around mid-gestation (156–159). It is on the basis of their ability to reconstitute the adult blood system that multipotent HSCs arising within the developing aorta have long been considered the precursors of adult HSCs. More recently, sophisticated lineage tracing and *in vivo* barcoding experiments have alternately suggested that many fetal HSCs and progenitors are transient, despite possessing multilineage capability (128, 160, 161). The abundance and diversity of progenitors underlying fetal hematopoiesis has driven an intense interest in defining their function and contribution to both the developing and adult immune systems.

Ongoing examination of how waves of fetal hematopoietic cell production contribute to adult immune compartments has begun to unravel two critical insights. First, distinct progenitors are responsible for generating parallel “waves” of immune cell production across development. Second, these distinct waves of immune cell production may contribute to functional heterogeneity within adult immune cell compartments. Precisely how developmental waves underlie functional heterogeneity in both normal and abnormal adult immune function is an area of active investigation. The best evidence to date to support the hypothesis that waves of developmental immune cell generation underlie heterogeneity of adult compartments comes from ongoing investigation of the origin of adult tissue-resident macrophages. Elegant studies using a range of fate-mapping and deletion models have revealed overlapping contribution from yolk sac, fetal liver, and adult progenitors across ontogeny (162–164). For example, dissection of the function of these distinct fetal-derived cell subsets in their resident tissues has revealed unique roles in electrical conduction in the heart (165), mammary gland remodeling (163), synaptic pruning (136), and surfactant clearance for lung alveoli (166). Indeed, recent work as further shown that fetal-derived macrophages may contribute uniquely to disease states, including cancer (167) and myocardial infarction (168). Illuminating the specific and precise contribution of macrophages derived from distinct progenitors and refining how specific waves contribute even greater functional heterogeneity within tissues is an ongoing effort that will further understanding of how early development contributes to normal and abnormal immune function across the lifespan.

### Hematopoietic Stem Cells as “Sensors” of Infection

**Figure 2:**
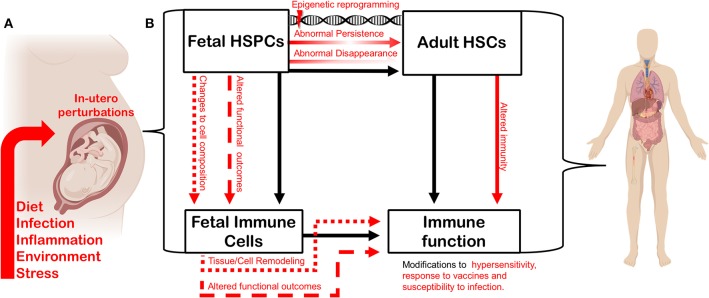
*In utero* perturbation shapes adult immunity by altering the establishment of the fetal hematopoietic and immune systems. **(A)** Factors such as maternal diet, infection, inflammation, environmental insult, and stress can influence fetal growth *in utero*. **(B)** *In utero* perturbations impact fetal hematopoietic stem and progenitor cells (HSPCs), thereby altering the composition or function of the fetal-derived immune cells they generate during development. The function and cell composition of fetal-derived immune cell compartments can also be impinged upon directly by *in utero* perturbation. Maternal perturbation can also drive changes (red arrows) to the adult hematopoietic stem cell (HSC) compartment. Transient fetal hematopoietic progenitors can fail to appear normally during ontogeny, or be induced to persist abnormally, driven by persistent epigenetic reprogramming. Such changes will ultimately impact heterogeneity and function of the adult HSC compartment. Perturbing the composition and function of the adult HSC compartment will alter adult immune output and the trajectory of the immune system. Similarly, as fetal-derived immune cells play critical roles in tissue development and homeostasis, disturbing their establishment or function can impact tissue-specific immunity and disease-risk across the lifespan. Figure created using Biorender.com.

The complexity of the developing hematopoietic and immune systems suggests that extrinsic inputs during fetal development could influence phenotypic outcomes for immune function in a variety of different ways, depending on *when* and *how* these inputs are interpreted. If subsets of immune cells that persist across the lifespan are produced *only* from transient fetal progenitors during specific windows of fetal development, the nature and timing of those extrinsic inputs will necessarily influence the trajectory of the immune system. Here we propose that extrinsic inputs could shape the trajectory of the immune system at the *progenitor* level (Figure 2). By perturbing the complicated waves of hematopoietic development that ultimately shape adult immune cell compartments, extrinsic inputs—including the type and extent of microbial exposure, and the maternal inflammatory environment shaped by distinct immune responses—could ultimately shift immune function in offspring. Superimposing the concept of a “critical period” over layered immune development provides a new perspective on how infection or inflammation during gestation might impact long-term immune function and drive hypersensitivity (Figure 2).

Recent investigation in adult hematopoiesis has illuminated the mechanisms by which adult HSCs can act as both direct sensors and drivers of the immune response during inflammation. In response to infection, the blood system rapidly produces short-lived myeloid cells required to counter infection. At the top of the hematopoietic hierarchy, adult HSCs have been documented to respond directly to systemic viral (169) and bacterial infections (170), as well as to a host of inflammatory cytokines, including type I and type II interferons (171–174), IL-1β (175), IL-27 (176), and TNF-α (173, 177), and specific TLR ligands (178, 179). In addition, adult HSCs have been reported to express a multitude of additional cytokine receptors, the functions of which in regulating HSC biology have yet to be investigated (180). With progressive exposure, adult HSCs lose self-renewal potential, face oxidative stress, and undergo metabolic changes that drive reprogramming of myeloid differentiation programs (181, 182). Recent evidence also suggests that specific progenitors within the heterogeneous adult HSC compartment differentially receive and drive the response to inflammation. Work by Essers and colleagues, for example, suggests that specific megakaryocyte-biased progenitors are induced upon acute inflammation to rapidly replenish platelets (173). How these rapid responses contribute to long-term changes in the adult HSC compartment remains to be determined.

In direct response to broad range of inflammatory stimuli, adult HSCs shift the trajectory of hematopoiesis by adopting a myeloid-biased output (181). Most recently, this response has been implicated as a driver of “trained innate immunity.” Whereas immune memory has typically been a distinct and critical feature of adaptive immunity, as noted earlier, trained innate immunity refers to the ability of the innate immune cells to evoke a stronger response to a non-specific stimulus following infection (84). The conundrum of trained innate memory is that most innate immune cells, such as monocytes, are short-lived, with a lifespan shorter than the timespan for which that “memory” has been observed. Recent work has shed light on one possible mechanism by which trained innate memory is “stored” by adult HSCs. Two recent publications have revealed that, in response to infection, hematopoietic progenitors specifically produce myeloid cells that have an enhanced response to subsequent infections. These persistent changes are driven by alterations in epigenetic profile and metabolism at the progenitors level (183, 184). These data provide additional support that the direct sensitivity and responsiveness of adult HSCs to inflammatory stimuli can redirect the long-term trajectory of the immune system.

### Beyond Congenital Infection—Maternal Inflammation Shapes Fetal Hematopoiesis

Considerably less is known about how fetal HSCs respond to inflammation. In light of growing appreciation that the adult HSC compartment is far more heterogeneous than previously recognized (185), the fetal HSC compartment is certain to be even more heterogeneous. For example, numerous progenitors with varying differentiation capacity have been identified within the last decade (128, 186–188). As the fetal HSC compartment is composed of heterogeneous, transient progenitors that continuously shift in space and time across development, defining their response to inflammation is a much more complicated feat. The study of fetal hematopoiesis and inflammation to date has been guided mostly by the concept of “sterile” inflammatory signaling—the requirement for transmission of pro-inflammatory signaling during HSC specification, but in the absence of any specifically defined source of inflammatory signal. Indeed, work in mice and zebrafish models has detailed the requirement for TNF receptors, and specific signaling pathways downstream of cytokine receptors, including Myd88 and NFkB, for HSC emergence [for recent review see (189)]. While recent work on HSC emergence has revealed the presence of pro-inflammatory macrophages in the developing aorta that may help drive endothelial to hematopoietic transition (190), there has generally been limited investigation of how specific infection or inflammatory signals during pregnancy might impact the fetal HSC compartment. Nonetheless, the capability of fetal HSCs to respond to inflammatory signals, and the responsiveness of the adult HSC compartment to such signals, certainly suggests that fetal hematopoietic progenitors could be responsive to infection and inflammation during gestation.

## Implications—A “Layered” Hygiene Hypothesis

We have reviewed evidence that extends the traditional notion of the hygiene hypothesis to include perturbations that occur *in utero*. The direct cellular mechanism driving training of the fetal immune system during early life is underscored by key characteristics unique to the fetal hematopoietic environment. HSCs can sense and respond to extrinsic stimuli by eliciting intrinsic changes to their function and output. While the specific mechanisms driving the fetal HSC response to such stimuli are unknown, distinct features, such as their transient nature and less quiescent state, leave fetal HSCs susceptible to environmental perturbation. Furthermore, fetal HSCs give rise to immune progeny that persist across the lifespan and contribute to adult immune function. The formation of a “layered” immune system, in which fetal-derived immune cells co-exist alongside adult bone marrow-derived immune cells, contributes to heterogeneity of adult immune cell compartments, particularly within tissues. Many fetal derived immune cells, including innate-like lymphocytes, are implicated in tissue-specific disease, and diseases of tolerance such as asthma and autoimmunity. Thus, impairment of fetal hematopoiesis and the establishment of fetal-derived immune cells can cause persistent changes to the trajectory of the immune system and disease susceptibility throughout the lifespan.

Because of the unique interface of the fetal-maternal environment, the concept of developmental perturbation can be extended to include a wide range of conditions or external stimuli that can occur during fetal development. In addition to pathogen exposure, these could include but are not limited to: maternal nutritional status, maternal obesity/underweight, maternal toxicant exposure, and maternal stress (Figure 2A). The mechanisms that define how the fetus responds to maternal perturbation are poorly understood, which opens the door to many more types of perturbations being involved in reprogramming immunity. While we have discussed a body of literature to demonstrate how infection influences offspring immunity, we still do not understand how inflammation during development directly impacts transient hematopoietic progenitors during fetal development and what the direct implications are for immunity in later life. Here we posit a few possibilities (Figure 2B): (1) Transient progenitors could disappear, and waves of immune cell production could be lost or altered. (2) Transient progenitors could be induced to persist abnormally, generating increased numbers of specific fetal-restricted cells. In both cases, the composition of adult immune cell compartments would be fundamentally shifted. (3) Another possibility is that progenitors could be cell-intrinsically re-programmed to produce functionally different immune cells. (4) Cell-intrinsic reprogramming of transient fetal progenitors could affect the make-up and function of the adult HSC compartment. All of these possibilities remain to be investigated using an established model of maternal infection, and all could have a significant influence on adult immunity and disease susceptibility.

The concept of *in utero* fetal immune “training” still leaves many questions to be answered. The distinct cellular mediators of fetal immune training by maternal inflammatory signals and the mechanisms by which maternal inflammation impacts fetal hematopoietic stem cell development have yet to be parsed out. Unpublished work in our lab has revealed the responsiveness of specific fetal hematopoietic stem and progenitor cells to maternal inflammation induced by TLR agonists, such as poly(I:C), and congenital infections, such as *Toxoplasma gondii*. These observations, along with the concepts reviewed above, underscore the need to expand the Th1/Th2 dichotomy and its role in early immune development through the lens of the hygiene hypothesis. We propose a broader understanding that accounts for the impact of early exposure to both hematopoietic stem cells and immune cells that arise during a critical window of development. Innate-like lymphocytes, immune cells that arise during a critical window of development, including innate-like lymhocytes. By impinging upon their establishment during fetal development, we train or manipulate the immune system in lasting ways.

## Future Questions Regarding Maternal—Fetal Immune Training

- What are the cellular mediators of fetal immune training by maternal inflammation?
- What is the mechanism by which maternal inflammation impacts fetal hematopoietic stem cell development?
- Does fetal immune training lead to fixed or transient changes to the immune system?
- Does the severity of maternal infection matter?
- Is the training generalized to all infections, or specific to certain microbes?
- Can the fetal immune system be trained (therapeutically) to lessen immune hypersensitivity disorders?

## Author Contributions

AB, AA, and KJ wrote and edited the manuscript.

### Conflict of Interest

The authors declare that the research was conducted in the absence of any commercial or financial relationships that could be construed as a potential conflict of interest.
